# Identifying risk factors for mortality among patients previously hospitalized for a suicide attempt

**DOI:** 10.1038/s41598-020-71320-3

**Published:** 2020-09-16

**Authors:** Riddhi P. Doshi, Kun Chen, Fei Wang, Harold Schwartz, Alfred Herzog, Robert H. Aseltine

**Affiliations:** 1 Division of Behavioral Sciences and Community Health, UConn Health, 263 Farmington Avenue, MC 6030, Farmington, CT 06030 USA; 2 Center for Population Health, UConn Health, Farmington, CT USA; 3 Department of Statistics, University of Connecticut, Storrs, CT USA; 4 Division of Health Informatics, Department of Healthcare Policy and Research, Weill Cornell Medical School, Cornell University, New York, NY USA; 5 Institute of Living, Hartford Healthcare, Hartford, CT USA; 6 Department of Psychiatry, University of Connecticut Health Center, Farmington, CT USA

**Keywords:** Health services, Psychiatric disorders

## Abstract

Age-adjusted suicide rates in the US have increased over the past two decades across all age groups. The ability to identify risk factors for suicidal behavior is critical to selected and indicated prevention efforts among those at elevated risk of suicide. We used widely available statewide hospitalization data to identify and test the joint predictive power of clinical risk factors associated with death by suicide for patients previously hospitalized for a suicide attempt (N = 19,057). Twenty-eight clinical factors from the prior suicide attempt were found to be significantly associated with the hazard of subsequent suicide mortality. These risk factors and their two-way interactions were used to build a joint predictive model via stepwise regression, in which the predicted individual survival probability was found to be a valid measure of risk for later suicide death. A high-risk group with a four-fold increase in suicide mortality risk was identified based on the out-of-sample predicted survival probabilities. This study demonstrates that the combination of state-level hospital discharge and mortality data can be used to identify suicide attempters who are at high risk of subsequent suicide death.

## Introduction

Suicide is a serious public health concern in the United States resulting in over 47,000 deaths each year^1^. Recent analysis indicates that the overall age-adjusted suicide rates have increased in the United States from 1999 to 2016, with increases reported among men and women and across all age groups^2^ despite the fact that suicide screening questions are a standard component of the clinical psychiatric interview. The ability to identify demographic and health event-related risk factors is critical to selected and indicated prevention efforts among those at elevated risk of suicide^3^.

The expanded use of electronic health records (EHR) in the US has stimulated efforts to identify patients at risk of suicide in different populations. A handful of studies using EHR and claims data have employed data mining and machine learning approaches to predict suicidal behavior and suicide mortality among patients in large healthcare systems^4–8^. Such studies have not only confirmed the importance of prominent clinical risk factors for suicide attempts and death identified in prior research (e.g., mental health diagnoses, particularly depressive disorders^9^, substance use disorders^10^, adverse childhood experiences^11^, HIV and sleep disorders^12^), but have also identified myriad other characteristics and features in their predictive algorithms that lead to greatly improved predictive accuracy compared to previous efforts^3,5,14^. In addition, follow-up of patients completing suicide risk assessments have found that predictive models applied to EHR data achieved higher sensitivity and specificity in identifying suicidal behavior than clinical assessments^15^. Similar findings were observed in a study utilizing veterans’ health data, providing additional evidence that, while clinicians may identify a state of risk using traditional clinical assessment techniques, predictive models are capable of identifying higher-risk patients who are missed during clinical assessments and are most likely to complete a lethal suicidal act^4,16^.

Despite the promise of using large healthcare databases to identify patients at risk of suicide, a critical challenge still remains: how to incorporate such models into clinical practice in diverse healthcare settings. Although there are many aspects to this challenge, including the alteration of clinical workflows, training providers and staff to respond appropriately to suicide risk^17^, and creating access to behavioral health treatment resources^18^, perhaps the most daunting among these is the limited data available to most healthcare providers. The rich datasets from which the most comprehensive and accurate algorithms have been generated are derived from large and sophisticated integrated delivery systems, health plans, and research networks^4,6,19^. Health system-wide medical records data of this nature are not, and may likely never be, available to the vast majority of healthcare providers in the US.

This study seeks to address this limitation by using available state-wide data to predict suicide mortality among patients previously hospitalized for a suicide attempt. There is strong evidence that individuals who have previously attempted suicide are at substantially elevated risk for subsequent death by suicide^20^, and risk stratification among patients in this vulnerable cohort could inform all aspects of care. The hospital discharge data used in this analysis are widely available in the US. Moreover, All-Payer Claims Databases (APCDs), which contain both inpatient and outpatient claims along with information related to pharmacy utilization, imaging, and laboratory data, are currently deployed in 27 states covering two-third of the US population^21,22^. If such data can be used to generate accurate models of subsequent suicide risk, their widespread availability would allow them to be employed in healthcare settings throughout the US.

## Methods

We analyzed data from adult patients (≥ 18 and ≤ 70 years) hospitalized for suicide attempts in Connecticut acute care hospitals between October 1, 2004–September 30, 2012. Patients under age 18 and over age 70 were omitted from the analysis due to concerns related to both bias and generalizability. When modeling the likelihood of death due to suicide, deaths from other causes may result in substantial bias in model estimation among elderly patients. In addition, many studies show that risk factors for suicide vary across different age groups, especially in children and elderly^23,24^. To address these concerns, we have limited the study to adult patients under 70 years of age at the time of their last admission. Patients with hospitalizations for suicide attempts were identified using both E-codes and other ICD-9 code combinations indicative of suicidal behaviors (supplemental digital content Table 1)^16–18^.

### Data sources

We obtained de-identified discharge data from the Connecticut Hospital Inpatient Discharge Database and mortality data indicating cause of death from the Office of Connecticut Medical Examiner. Both contained a unique identifier within each dataset, although in the case of the discharge data this identifier was only consistent within hospitals. To detect multiple admissions for the same patient across hospitals and to integrate the hospitalization and mortality data, a unique patient identifier was generated using the patient’s date of birth, sex, race, and ethnicity, based on previous work indicating that such characteristics can be used to accurately link individuals across databases^25^. For each patient, multiple admission records were aggregated to the time of the most recent nonfatal attempt. Patients who died during their only hospitalized attempt were excluded (~ 1%). Of the 571 matches between the 2 datasets, 93.7% were unique; the remaining 6.3% involved the linkage of a hospitalization for suicide attempt with multiple suicide death events. For these cases the time of death was randomly assigned from one of the two matching records.

The Connecticut Department of Public Health Human Investigations Committee approved this research project. This project was ruled as non-human subjects research by the University of Connecticut Health Center Institutional Research Board. This research involved no interaction with human subjects.

### Measures

Our analysis included sociodemographic variables including patient’s age, sex, race, and Hispanic ethnicity; the frequency and duration of hospitalizations including number of suicide-related admissions, and average length-of-stay across admissions; primary and secondary ICD-9 diagnosis codes, procedure codes, and discharge status. The first three digits of the ICD-9 codes were used as indicator variables. The primary outcome variable was time to death by suicide.

### Statistical analysis

For each patient, the follow-up period for survival modeling began at the most recent nonfatal hospitalization for suicide attempt and continued until death or the end of the study period on September 30, 2012. Since there were a large number of factors (> 400), a marginal variable screening procedure was performed. We tested the association of each variable with survival time using a Cox proportional hazard regression model that controlled for race/ethnicity, sex and age. Variables with p-value less than 0.05 were kept for predictive modeling analysis. Subsequently, a stepwise Cox model was used for variable selection and model estimation, with the main-effects and two-way interactions of variables passing the screening included as candidate predictors. The final model was adjusted to include both main effects whenever an interaction term was selected. We tested the proportional hazard assumption for each selected variable as well as for the overall model; all tests indicated that the assumption was not violated (*p* = 0.34). For ease of interpretation, we chose the estimated 5-year survival probability as the risk measure.

To objectively determine a survival probability cut-off to identify high-risk patients and to assess the predictive power of the Cox model, we conducted an out-of-sample random-splitting procedure. The data was randomly split into 80% for training and 20% for testing. The Cox model was fitted using the training data, and the fitted model was then used to estimate the 5-year survival probabilities of the patients in the testing data. A high-risk group was identified as patients whose estimated probabilities exceed certain cut-off value. For each candidate cut-off, we computed (1) the risk ratio between the high-risk group and the testing cohort, defined as the ratio for observed deaths within 5-years, and (2) the relative size of the high-risk group among the test subjects. The accuracy of risk classification was then assessed by the Area Under the ROC Curve (AUC). This random-splitting procedure was repeated 300 times, and results were averaged.

### Conference presentation

"Novel Predictors Of Suicide Mortality: A Statewide Analysis" presented at the Mental Health Services Research Conference organized by the National Institute of Mental Health, Washington, D.C., August 1–4, 2018.

## Results

### Risk factor identification

Table 1 presents the composition of the study population by age group, sex, race/ethnicity and median household income of the patient’s residential zip code. We observed 571 suicide deaths among 19,057 patients hospitalized for suicide attempts by the end of the study period. Men, non-Hispanic Whites, those aged 45–59 years, and those living in zip codes with higher median incomes were at highest risk for suicide mortality. Table 2 presents further information on the method used for the (most recent) prior suicide attempt, the number of previous attempts, and psychiatric diagnoses at the prior attempt. Multiple previous attempts were associated with subsequent mortality, and while more than half of all patients had a mental health diagnosis, there was no association between these mental health conditions and death by suicide ( χ^2^ test; *p* = 0.15).

**Table 1:** Characteristics of the study population.

	All patients with prior attempt		Prior attempters dying by suicide within study period		Prior attempters not dying by suicide within study period	
	N	%	N	%	N	%
Total	19,057	100.0	571	3.0	18,486	97.0
**Age group**						
18–29	5,577	29.3	109	19.1	5,468	29.6
30–44	6,382	33.5	214	37.5	6,168	33.4
45–59	5,881	30.9	227	39.8	5,654	30.6
60 +	1,217	6.4	21	3.7	1,196	6.5
**Sex**						
Male	8,840	46.4	393	68.8	8,447	45.7
Female	10,217	53.6	178	31.2	10,039	54.3
**Race/ethnicity**						
Black	1,957	10.2	8	1.4	1,949	10.5
Asian	128	0.7	1	0.2	127	0.7
Hispanic	2,472	13.0	4	0.7	2,468	13.4
White	13,909	73.0	557	97.5	13,352	72.2
Other	589	3.1	1	0.2	588	3.2
**Median household income (by residential zip code)**						
	Mean	SD	Mean	SD	Mean	SD
	65,002	26,479	71,512	27,563	64,801	26,421

**Table 2:** Characteristics of the study population.

	All patients with prior attempt		Prior attempters dying by suicide within study period		Prior attempters not dying by suicide within study period	
	N	%	N	%	N	%
**Number of suicide attempts**						
1	16,309	85.6	459	80.4	15,850	85.7
> 1	2,748	14.4	112	19.6	2,636	14.3
**Method of suicide attempt**						
Poisoning	14,542	76.3	431	75.5	14,111	76.3
Hanging	279	1.5	18	3.2	261	1.4
Firearms	123	0.6	1	0.2	122	0.7
Cutting	3,235	17.0	99	17.3	3,136	17.0
Jumping	148	0.8	4	0.7	144	0.8
Drowning	5	0.0	0	0.0	5	0.0
Other	725	3.8	18	3.2	707	3.8
**Psychiatric diagnosis**						
Mood disorders	10,356	54.3	316	55.3	10,040	54.3
Psychotic disorders	343	1.8	14	2.5	329	1.8
Anxiety disorders	6	0.0	0	0.0	6	0.0
Substance abuse disorders	1,295	6.8	34	6.0	1,261	6.8

We present data from our analyses of the risk factors for later suicide mortality in Table 3. This table combines the results of two separate analyses. First, since there were a large number of potential predictive factors (> 400), a marginal variable screening procedure was performed. Table 3 (model 1) presents the marginal effects of 28 risk factors that were significantly associated with suicide death after controlling for age, sex and race. The significant marginal effects and their 2-way interactions were then used to build a Cox proportional hazards model, with the final terms selected using a stepwise estimation method. Detailed information about the coefficients, confidence intervals and significance levels for all the factors in the selected Cox proportional hazards model are included in Table 3 (model 2), although we caution against the interpretation of individual parameter significance in this specification of the model since the computation of the p-values does not account for the uncertainty in predictor selection.

**Table 3:** Results from marginal variable screening analysis and Cox proportional hazards model predicting time to suicide death among suicide attempters.

		Marginal screening results				Cox proportional hazards results ^d^			
	n ^c^	Hazards ratio	CI lower	CI upper	*p* value	Exp (coef)	CI lower	CI upper	*p* value
**Demographics**									
Age^a^	–	–	–	–	–	1.2	1.1	1.3	< .0001
Sex^a^ (Male = 1)	–	–	–	–	–	2.7	2.3	3.3	< .0001
Race (White) ^a^	–	–	–	–	–	24.5	11.6	51.9	< .0001
Median household income ($65,002)	N/A	1.1	1	1.2	0.047	1.1	1	1.2	0.042
**Contextual features of the prior attempt**									
Multiple suicide attempts	2,748	1.1	1.1	1.2	0.001	1.1	1	1.1	0.172
Discharged/transferred to a psychiatric hospital or psychiatric distinct part unit of a hospital	3,353	1.5	1.2	1.8	0.001	7.3	2.4	21.8	< .0001
Discharged/transferred to another type of institution for inpatient care	2,197	1.2	1	1.5	0.035	1.2	0.9	1.5	0.150
Other persons seeking consultation	166	2.6	1.4	4.8	0.003	2.4	1.3	4.4	0.008
**Procedures**									
Non-operative intubation and irrigation ^b^	2,619	1.3	1.1	1.6	0.009	–	–	–	–
Other operations on bones, except facial bones	37	2.7	1	7.3	0.046	18.7	1.38	253.5	0.028
Operations on esophagus	23	5.8	2	14.5	0.001	5	1.9	13.6	0.001
Operations on penis	9	8.4	2.1	33.7	0.003	8.2	2	34.4	0.004
Operations on tongue	9	8.3	2	33.2	0.003	6	1.1	32.5	0.037
Operations on the breast ^b^	7	7.2	1	51.6	0.048	–	–	–	–
Aortic and heart assistant procedures except pulsation balloon without MCC	12	4.5	1.1	18	0.034	6.6	0.9	49.7	0.067
**Diagnoses/Methods of suicide attempt**									
Suicide attempt by hanging	299	1.9	1.2	3	0.009	1.6	0.9	2.6	0.080
Accidental poisoning by drugs, medicinal substances, and biologicals	2,758	0.7	0.6	1	0.025	0.6	0.4	0.8	0.002
Injury undetermined whether accidentally or purposely inflicted	2,287	0.6	0.5	0.8	0.002	0.7	0.5	0.9	0.005
Other psychosocial circumstances	2,219	1.5	1.2	1.9	0.001	1.4	1.01	1.8	0.023
Poisoning and toxic effects of drugs with major comorbid condition (MCC)	2,209	1.4	1.1	1.8	0.012	1.3	0.99	1.7	0.084
Toxic effects of alcohol ^b^	1,378	1.4	1	1.8	0.025	–	–	–	–
Other and unspecified disorders of back	849	0.6	0.4	0.9	0.027	0.4	0.2	0.8	0.005
Poisoning by primarily systemic agents	813	1.5	1.1	2.2	0.023	1.2	0.8	1.9	0.303
Other nonorganic psychoses	329	1.8	1.1	3.1	0.028	1.5	0.9	2.8	0.141
Accidental poisoning by other solid and liquid substances, gases, and vapors	276	1.9	1.1	3.2	0.015	1.7	1	3	0.051
Organic sleep disorders	202	2.1	1.2	3.8	0.010	2.1	1.2	3.7	0.013
Open wound of other and unspecified sites except limbs	169	2.0	1.1	3.7	0.031	1.3	0.6	2.8	0.554
Human immunodeficiency virus	123	2.1	1	4.5	0.048	1.8	0.9	4	0.121
Multiple sclerosis	97	3.2	1.6	6.5	0.001	0.4	0	4.5	0.478
Seizures without MCC	16	5.3	1.3	21.1	0.019	4.3	1.1	17.3	0.042
Disorders of the pancreas except malignancy without MCC ^b^	15	5.0	1.3	20.2	0.023	–	–	–	–
**Interactions**									
Multiple sclerosis with other nonorganic psychoses	–	–	–	–	–	63.4	6.8	588.3	< .0001
Other nonorganic psychoses with open wound of other and unspecified sites except limbs	–	–	–	–	–	15.1	1.7	137.5	< .0001
Other psychosocial circumstances with other operations on bones, except facial bones	–	–	–	–	–	14.8	1.5	146.6	< .0001
Hanging with other and unspecified disorders of back	–	–	–	–	–	9.8	2.1	46.6	0.004
Number of visits with aortic and heart assistant procedures except pulsation balloon without MCC	–	–	–	–	–	9.1	2	40.1	0.004
Accidental poisoning by drugs, medicinal substances, and biologicals with open wound of other and unspecified sites except limbs	–	–	–	–	–	8.3	1	71.6	0.053
Accidental poisoning by other solid and liquid substances, gases, and vapors with poisoning by primarily systemic agents	–	–	–	–	–	7	1.4	34.7	0.016
Accidental poisoning by drugs, medicinal substances, and biologicals with other and unspecified disorders of back	–	–	–	–	–	3.4	1.3	9.1	0.016
Poisoning and toxic effects of drugs with MCC with poisoning by primarily systemic agents	–	–	–	–	–	2.3	0.9	5.7	0.076
Operations on tongue with median income	–	–	–	–	–	2.2	0.9	5.8	0.093
Age with accidental poisoning by drugs, medicinal substances, and biologicals	–	–	–	–	–	0.8	0.6	1.1	0.125
White race with discharged/transferred to a psychiatric hospital or psychiatric distinct part unit of a hospital	–	–	–	–	–	0.2	0.1	0.5	0.001
Multiple sclerosis with median income	–	–	–	–	–	0.2	0.1	0.9	0.042
White race with other operations on bones, except facial bones	–	–	–	–	–	0	0	0.4	0.005

^a^Age, sex, and race were not included in the marginal screening analysis.

^b^These procedures/diagnoses were not selected for inclusion in the final Cox proportional hazards model.

^c^This column presents the number of patients in the analysis with this characteristic.

^d^We caution against the interpretation of individual parameter significances in this specification of the model since the computation of the *p*-values does not account for the uncertainty in predictor selection.

Results presented in Tables 3 and 4 (model 2) indicated that the socio-demographic factors positively associated with suicide deaths included being male, older age, White race and higher median household income. Diagnosis codes including organic sleep disorders, seizure without major comorbidities, other psychosocial circumstances and other persons seeking consultations were positive predictors of suicide deaths. Diagnostic codes related to method of suicide attempt including accidental poisoning by drugs, medicinal substance and biological, injury undetermined whether accidentally or purposely inflicted and other unspecified disorders of back were also significant predictors of suicide deaths. Many medical procedures that were likely due to the method and severity of the suicide attempt, such as procedures on the esophagus, suture of the tongue, and surgeries on bones particularly tibia and fibula, were positive predictors of suicide deaths. In addition, suicide attempts accompanied by operations on the penis were associated with subsequent suicide death.

**Table 4:** Summary of risk and protective factors associated with death by suicide among previous attempters.

Demographic factors	Contextual features of the prior attempt
Older age	Multiple suicide attempts
Male sex	Discharged/transferred to a psychiatric hospital or psychiatric unit of a hospital
White race	Discharged/transferred to another type of institution for inpatient care
Higher median household income	Others persons seeking consultation

Procedures	Methods of attempt and associated diagnoses/comorbidities
*Non-operative intubation and irrigation*	Accident poisoning by drugs, medicinal substances, and biologicals (−)
Other operations on bones, except facial bones	Poisoning and toxic effects of drugs with major comorbid condition (MCC)
Operations on Esophagus	*Toxic effects of alcohol*
Operations on penis	Poisoning by primarily systematic agents
Operations on tongue	Accidental poisoning by other solid and liquid substances, gases, and vapors
*Operations on breast*	Open wound of other and unspecified sites except limbs
Aortic and heart assistant procedures except pulsation balloon w/o MCC	Suicide attempt by hanging
	Injury undetermined whether accidentally or purposely inflicted (−)
	Other and unspecified disorders of back (−)
	Other nonorganic psychoses
	Other psychosocial circumstances
	Organic sleep disorders
	Human immunodeficiency virus
	Multiple sclerosis
	Seizures w/o MCC
	*Disorders of the pancreas except malignancy w/o MCC*

Entries in italics were not selected in the final Cox proportional hazards model. A minus sign (−) indicates a protective factor in which this characteristic was associated with lower risk of subsequent mortality.

Several significant interactions terms were also selected into the final Cox model. Patients who were discharged or transferred to a psychiatric hospital or a psychiatric unit of the same hospital had higher suicide risk, and this effect was much stronger for non-Whites compared to Whites. A similar interaction was observed between race and operations on the bone, with the sign of this effect indicating a lesser impact of such operations among Whites. Among patients with multiple suicide attempts and aortic and heart assistant procedures, the mortality risk was higher. Coexistence of multiple sclerosis with other non-organic psychoses also increased the risk of later suicide death. Non-organic psychoses interacted with open wound of other and unspecified sites to increase the risk of suicide deaths. A number of other interactions were observed among diagnostic codes related to methods of suicide including poisoning, back disorders, open wounds and hanging.

### Predictive performance

**Figure 1:**
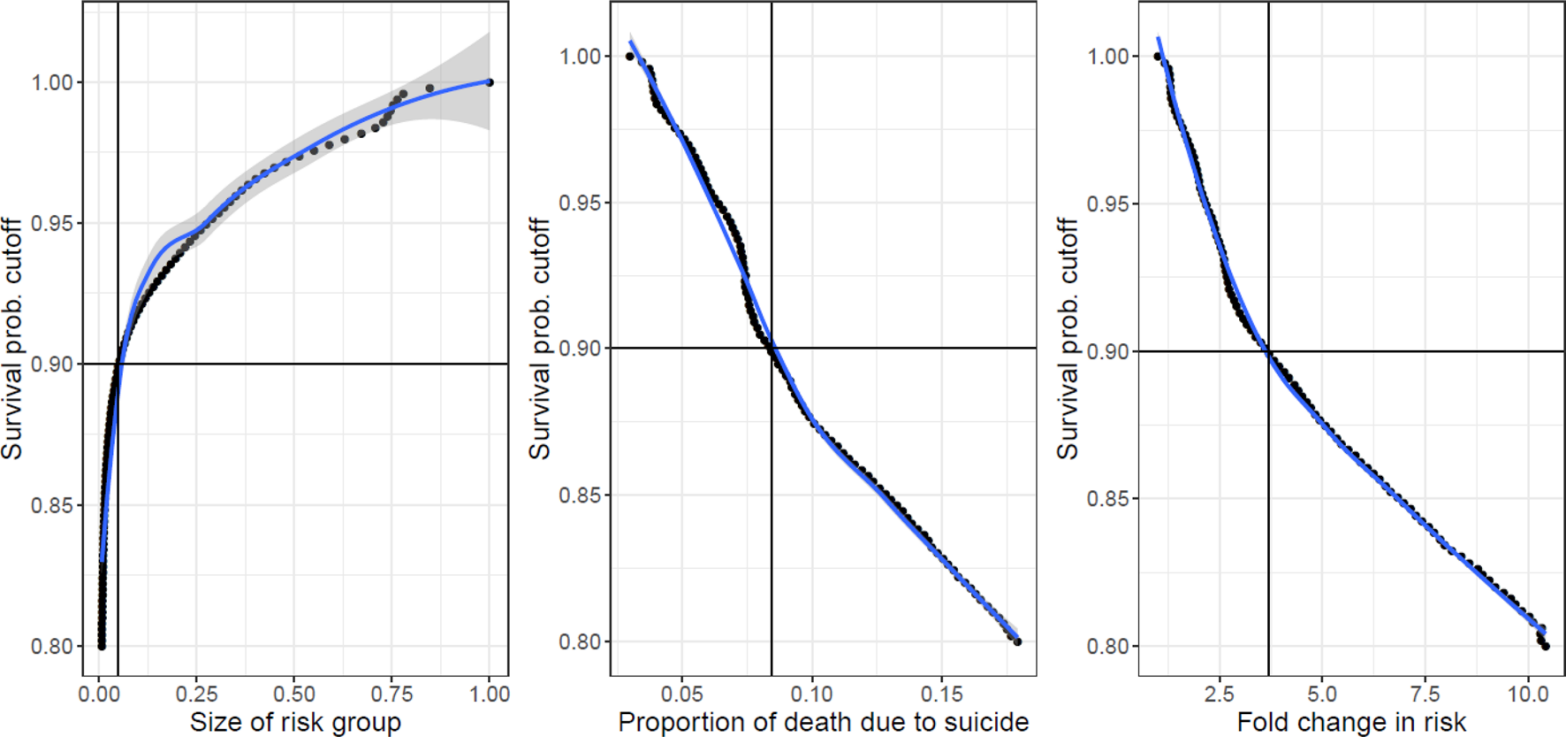
The probability cutoff, the size of the high-risk group relative to the general cohort, and the increase in suicide risk.

The estimated 5-year survival probability was used as a risk measure to identify high-risk patients. Figure 1 demonstrates the relationships between the probability cut-off, the size of the high-risk group relative to the general cohort, and the increase in suicide risk, based on the out-of-sample random splitting procedure. As expected, the lower the cut-off value, the higher the overall risk level of the identified high-risk group, and the smaller the size of the high-risk group. Our results show that if the high-risk group is defined as consisting of subjects whose 5-year survival probabilities were smaller than 0.90, then it equaled 4.9% [90% CI: (3.9, 5.8)] of the general cohort, and the risk of death in this group was on average 3.71 (90% CI: [2.371, 5.435]) times that in the general cohort.

**Figure 2:**
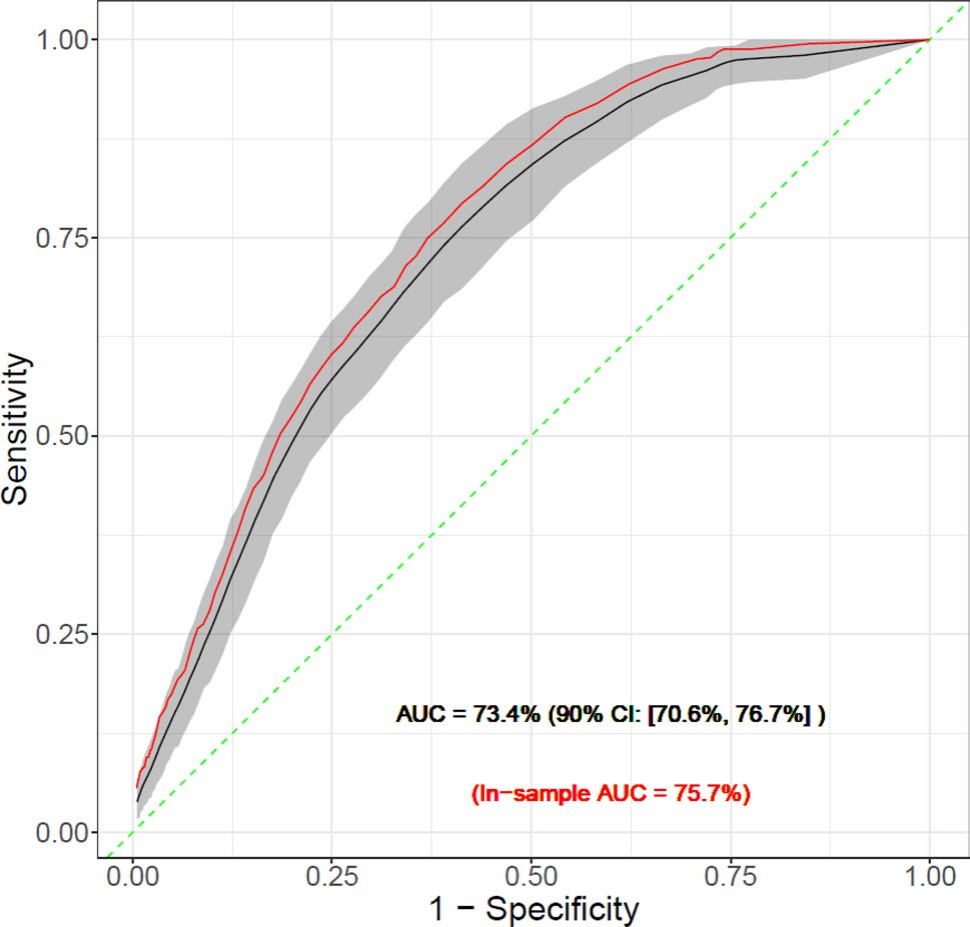
Out-of-sample mean ROC curve and its 90% confidence bands computed from the random splitting procedure.

In Fig. 2, we present the out-of-sample mean ROC curve and its 90% confidence bands computed from the random splitting procedure. With 80% sensitivity our model can achieve 55.2% specificity (90% CI:[48.9, 61.7]), with 50% sensitivity our model can achieve 79.6% specificity (90% CI:[75.8, 83.3]), and the mean AUC is 73.4% (90% CI:[70.6, 76.7]). The positive predictive value (PPV) is 7.1% [90% CI: (6.1%, 8.5%)] with a sensitivity of 0.5 and is 5.26% [90% CI: (4.6%, 6.0%)] with a sensitivity of 0.8, making this one of the best performing suicide prediction models published to date^19^.

## Discussion

In this study, we used widely available healthcare data to develop an interpretable model to predict suicide mortality following a prior attempt. In addition to augmenting the small but growing body of research on suicide mortality in high-risk populations^5,20–22^, our findings are relevant to the substantial portion of eventual suicides who first come to the attention of mental health clinicians through a prior suicide attempt, and show that clinical and contextual features from the prior attempt can be harvested from data to create a predictive model with good sensitivity and specificity. In fact, in comparison to other suicide mortality prediction models^26^, the sensitivity, specificity, and positive predictive value reported above makes our model among the best performing suicide mortality models published to date.

Several novel risk factors emerged from our analysis, including non-malignant pancreatic disorders, medical procedures associated with the prior attempt that could be indicative of the severity of injury, such as non-operative intubation and irrigation, aortic and heart assistant procedures, operations on bones, and operations on the penis. Regarding markers of injury severity, the clinical management of highly lethal suicide methods such as hanging often involves aggressive resuscitation and treatment of post-anoxic brain injury requiring intubation of attempters^27,28^. In case of suicide attempts by jumping, research evidence has demonstrated that lethal attempts have a very high probability of fractures of the upper limb which drastically increase surgical and inpatient workload due to the need for operations on bones^29^. Although operations on the penis were observed in a very small number of cases, they were associated with an eight-fold risk of later suicide death and may be indicative of the very high risk of suicide in patients with severe psychosis, which has been associated with genital mutilation^26^.

While the identification of specific markers for suicide mortality in psychiatric practice is important, the major contribution of this analysis lies in using data available in healthcare settings to identify the highest risk members of this already high-risk cohort. The cohort of patients hospitalized for suicide attempts included in this analysis accounted for approximately 25% of all suicide deaths among adults in Connecticut from 2005–2012 (571 out of 2,219)^30^. Our AUC analysis showed that 50% of the deaths in this cohort occurred among 21% of patients deemed at highest risk based on our model. In other words, our model identified approximately 4,000 high-risk patients, of which nearly 300 would die by suicide within 5 years of their attempt. Also, because all of the information used in the model is available at the time of discharge following a prior attempt, the elevated risk of particular patients could be incorporated into discharge planning and care transitions, and inform long-term approaches to treatment making it more implementable than past modeling efforts.

In terms of limitations, our analysis used discharge data; access to a broader array of healthcare data, such as ambulatory visits or pharmacy data could improve the predictive power of the model. At the time of this analysis we were limited to a combined dataset linking discharges and mortality through 2012. While we had no direct way of assessing the accuracy of the linkages in the absence of a shared unique identifier present in both databases, there are several reasons to have confidence in the accuracy of linkages derived from the demographic characteristics we used for matching. Research has shown that basic demographic characteristics such as those used in our analysis can be successfully used to connect individuals across very large, generic databases^25^, and in our case we had the additional advantage of linking very particular, related databases. Since both datasets were related to suicidal behavior, the accuracy of any match was likely to be much higher than what it would have been for a generic population of a larger size. Second, the potential for mismatches was limited by the presence of a unique identifier in both databases (noting that in the case of the hospital dataset this was only true *within* hospitals). Third, the datasets contained all hospitalizations and all suicide deaths in the state; absent data errors (such as an incorrect date of birth) or hospitalizations/deaths occurring outside the state, there was very limited potential for incomplete data in either database to result in matching failures. Finally, unless matching errors were systematic, their effect would be to introduce noise into the analysis. The fact that our final model was highly interpretable and had good out-of-sample predictive power indicates that the linkage between hospitalization and death records was highly accurate.

An additional limitation is that the relatively small number of suicide deaths precluded investigation of alternative model specifications, particularly sex-specific risk models. Finally, this study is limited to hospitalizations and deaths within Connecticut, which has one of the lowest suicide rates and is one of the most affluent states in the US. However, Connecticut’s proportion of non-White residents makes it slightly more diverse than the nation as a whole^31^.

Despite these limitations, the results from this study have major implications for clinical practice. Although there is robust literature showing that a prior attempt is a very strong risk factor for subsequent suicidal behavior and death by suicide, our work has shown that the risk of later mortality is confined to a relatively small subset of these patients, thus increasing opportunities to focus attention and resources on a smaller and more manageable patient population. In addition, it is important to emphasize that deploying suicide risk algorithms during the psychiatry consults at the time of hospitalization may substantially enhance clinical suicide risk assessments.

## Supplementary information

Supplementary Tables.

## Data Availability

The data used in this study were obtained from the Connecticut Department of Public Health and the Office of the Connecticut Medical Examiner under terms that do not permit the authors to disclose or make this information publicly available. Requests for access to these datasets must be made directly to these agencies.
