# Supplementary material for: Identifying risk factors for mortality among patients previously hospitalized for a suicide attempt
Source: Sci Rep. 2020 Sep 16;10:15223. doi: 10.1038/s41598-020-71320-3 (PMC7495431; doi:10.1038/s41598-020-71320-3)
Supplement: Supplementary file 1 — Supplementary Tables. [file 41598_2020_71320_MOESM1_ESM.docx]

**Identifying Risk Factors for Mortality among Patients Previously Hospitalized for a Suicide Attempt**

Riddhi P Doshi MBBS, PhD^1,2^, Kun Chen, PhD^2,3^, Fei Wang, PhD^4^, Harold Schwartz, MD^5,6^, Al Herzog, MD^5^, Robert H Aseltine Jr., PhD^1,2,3^ *

**Supplemental table 1:** Diagnostic (ICD9 CM) codes used to suicide-related admissions

| **Code Type** | **ICD-9 codes** |
| --- | --- |
| Suicide Attempt | E950-E958 (intentional self-harm) |
| Possible Suicide Attempt | E980-E988 (undetermined intent) |
| Suicide V-Code | V62.84 (suicide ideation) --AND SAME VISIT-- 870-899, 960-989 |
| Suicide Algorithm | 881, 960-979, 980-989, 994.7 ----AND SAME VISIT---- 293.83, 296.20-296.36, 296.82, 296.90, 298.0, 300.4, 309.0-309.1, 311, 296.00-296.06, 296.1-296.14, 296.40-296.89, 296.99, 301.13, 301, 290.8-290.9, 295, 297, 298.1-298.9, 299, 301.20-301.22, 780.1, 309.2-309.9 |

**Supplemental table 2:** Diagnostic codes for screening of mental health comorbidities including mood, psychotic, anxiety and substance abuse disorders

| **Condition** | **ICD9 diagnostic codes** |
| --- | --- |
| **Mood disorders** | 293.83, 296.2, 296.3, 296.90, 296.99, 298, 300.4, 301.12, 309.0, 309.1, 311, 296.0, 296.1, 296.4-8 |
| **Anxiety disorders** | 300.00-300.02, 300.09, 300.1, 300.21, 300.22, 300.23, 300.29 |
| **Psychotic disorders** | 295.0-295.9, 297.0-297.3, 297.8, 297.9 |
| **Substance abuse disorders** | 291, 292, 303, 304, 305 |
